# Inflammatory Bowel Disease and Skin Cancer: An Assessment of Patient Risk Factors, Knowledge, and Skin Practices

**DOI:** 10.1155/2016/4632037

**Published:** 2016-02-29

**Authors:** Jessica N. Kimmel, Tiffany H. Taft, Laurie Keefer

**Affiliations:** ^1^Department of Medicine, Division of Gastroenterology and Hepatology, Northwestern University School of Medicine, Arkes Family Pavilion Suite 1400, 676 North Saint Clair Street, Chicago, IL 60611, USA; ^2^Icahn School of Medicine, Mount Sinai Medical Center, Susan and Leonard Feinstein IBD Center, 17 East 102nd Street, 5th Floor, New York, NY 10029, USA

## Abstract

*Objective*. Patients with inflammatory bowel disease (IBD) are at increased risk from skin cancer. Aims include assessing IBD patients' risk factors and knowledge of skin cancer and current skin protection practices to identify gaps in patient education regarding skin cancer prevention in IBD.* Methods*. IBD patients ≥ 18 years were recruited to complete an online survey.* Results*. 164 patients (mean age 43.5 years, 63% female) with IBD (67% Crohn's disease, 31% ulcerative colitis, and 2% indeterminate colitis) were included. 12% (*n* = 19) of patients had a personal history and 34% (*n* = 55) had a family history of skin cancer. Females scored better on skin protection (16.94/32 versus 14.53/32, *P* ≤ 0.03) and awareness (35.16/40 versus 32.98/40, *P* ≤ 0.03). Patients over 40 years old scored better on prevention (17.45/28 versus 15.35/28, *P* = 0.03). Patients with skin cancer scored better on prevention (20.56/28 versus 15.75/28, *P* ≤ 0.001) and skin protection (21.47/32 versus 15.33/32, *P* ≤ 0.001). 61% of patients recognized the link between skin cancer and IBD.* Conclusions*. The majority of IBD patients are aware of the link between skin cancer and IBD; however, skin protection practices are suboptimal. This emphasizes the role of healthcare professionals in providing further education for skin cancer prevention in the IBD population.

## 1. Introduction

Skin cancer is the most common form of cancer in the United States and causes significant morbidity and mortality [1]. Inflammatory bowel disease (IBD) is a chronic autoimmune condition that is associated with increased risk of development of skin cancer. Proposed mechanisms predisposing IBD patients to skin cancer include chronic inflammation, cellular damage, and underlying immune dysfunction leading to altered tumor surveillance [2–4].

Use of immunosuppressants in IBD patients has been shown to lead to a 4–7-fold increased risk of skin cancer and approximately half of IBD patients are exposed to these medications within 5 years of diagnosis [3]. Specifically, the use of biologic and immunomodulating agents increases skin cancer risk [2–6]. Similarly, immunosuppression has been shown to accelerate the development of skin cancer in transplant patients; thus, routine skin exams are recommended [3, 4]. According to the United States Preventive Service Task Force, there is insufficient evidence to assess the risk versus benefit for general skin cancer screening [7]. In addition, there is a lack of data to support that early detection of skin cancer reduces morbidity and mortality [5]. However, patients with IBD have different risk factors for skin cancer development compared to the general population. Thus, these skin cancer screening recommendations may not be applicable to IBD patients. Despite the lack of specific standardized guidelines for screening, there is general consensus among gastroenterologists that IBD patients should protect themselves from the sun and that annual skin cancer surveillance should be considered, especially for patients on biologic and immunomodulating agents [5, 8, 9].

Thus, it is important for IBD patients to be aware of their risk of skin cancer and to adopt preventive strategies for modifiable risk factors. The aims of this study are to assess IBD patients' risk factors for and knowledge of skin cancer. In addition, we will assess patients' current skin protection practices. We anticipate that this survey will help to identify gaps in patient education regarding skin cancer prevention in the IBD population.

## 2. Methods

### 2.1. Patients

Patients 18 years of age or older with a diagnosis of inflammatory bowel disease were eligible for inclusion in the study. Due to the study design, a diagnosis of IBD was via self-report. Patients were recruited via email, by online sources, and by healthcare providers in gastroenterology offices at Northwestern Medicine. All subjects gave informed, signed consent prior to study registration. Eligible patients who agreed to participate were forwarded to an online, confidential, anonymous survey. Data was collected using Adobe FormsCentral. Study design was approved by the Northwestern University Institutional Review Board.

### 2.2. Survey Design

A cross-sectional research questionnaire queried patients about baseline demographics and IBD-related information, including medications, complications, and extent of their IBD. In addition, study participants were questioned about their individual skin cancer risk factors, sun exposure and skin protection practices, behaviors in the event that they identified a mole, and general skin cancer knowledge. Topics addressed in this study were compiled after critical review of published literature on skin cancer and its risk factors and included a compilation of questions in formats derived from other validated skin cancer assessments [6, 10–16]. Answers were formatted with Likert scales, true/false, and multiple-choice responses (Table 1). Questions were reviewed by dermatologists prior to administration.

### 2.3. Statistical Analyses

All responses were exported from the online system into SPSS v.22 for analysis. Data were tested for normal distribution. Descriptive statistics (percentages, mean (SD)) analyzed the demographic and clinical characteristics of the study sample. Scores for each skin cancer category were summed to produce total scores for skin cancer prevention (out of 28), awareness (out of 40), knowledge (out of 4), protection (out of 32), and exposure (out of 24). Each score was compared by demographic and clinical variables via a series of independent samples* t*-tests and one-way analysis of variance (ANOVA). Relationships between continuous variables were evaluated via Pearson's correlations while categorical variables were analyzed via Chi Square. Statistical significance was set to *P* ≤ 0.01 for *t*-tests and ANOVA to control for type I error due to multiple comparisons.

## 3. Results

A total of 164 patients with inflammatory bowel disease completed the online survey. Patient demographics are reported in Table 2. The mean age of participants was 43.5 years and 63% of patients were female. The majority of study participants were Caucasian (94%, *n* = 153) and non-Hispanic (98%, *n* = 161). Crohn's disease was reported in 67% of patients, ulcerative colitis in 31%, and indeterminate colitis in 2%. Ninety-five percent of patients were currently receiving IBD treatment at the time of survey response. Approximately two-thirds (*n* = 105) of patients reported either current or past treatment with immunomodulators (which include Imuran/Azathioprine and 6-mercaptopurine) and approximately two-thirds with biologics (*n* = 103).

Sunburn and skin cancer history were assessed and reported in Table 3. Twelve percent of patients (*n* = 19) reported a personal history of skin cancer, of which 7% were basal cell carcinoma, 2% squamous cell carcinoma, 1% melanoma, and 4% multiple types of skin cancer. Additionally, 34% (*n* = 55) reported having a first-degree relative with skin cancer. Sixty-two percent (*n* = 102) of patients had three or more episodes of bad sunburns. The majority of patients (70%) had seen a dermatologist, with 35% of patients receiving a full skin exam once per year, 30% less than once per year, and 24% having never received a skin exam. Half of patients (51%, *n* = 84) were self-referred to a dermatologist; gastroenterologists referred 13% of patients for dermatology consultation and primary care referred 17%.

Patient responses to standardized questions of skin cancer prevention, awareness, knowledge, protection, and sun exposure are reported in Table 1. Average scores for skin cancer prevention, awareness, knowledge, skin protection, and sun exposure were 16.24/28, 34.34/40, 2.93/4, 16.1/32, and 6.15/24, respectively. Patients over 40 years of age scored higher on prevention (17.45/28) compared to patients 40 years old or younger (15.35/28, *P* = 0.03). Females scored higher on skin protection (16.94/32 compared to 14.53/32 for males, *P* = 0.02) and awareness (35.16/40 compared to 32.98/40 for males, *P* = 0.03, Table 4). There were no statistically significant differences in scores between participants with or without exposure to immunomodulators. There was a difference in knowledge scores between those currently treated with biologic agents (3.01/4) compared to those with prior biologic exposure (2.91/4) or no exposure (2.85/4, *P* ≤ 0.01). Additionally, those who had a personal history of skin cancer scored better on both skin protection (21.47/32) and prevention (20.56/28) questions compared to those without a personal history (15.33/32, 15.75/28, resp., *P* ≤ 0.001). In addition, those with a family history scored higher on skin protection questions (18.46/32) compared to those without a first-degree relative with skin cancer (14.03/32, *P* ≤ 0.001). When asked if IBD and its treatment increase the risk of skin cancer, 61% of patients agreed with the statement and 38% responded neutrally or disagreed (Table 5); patients currently or previously treated with immunomodulators scored higher than those who were not on these medications (*P* = 0.000).

## 4. Discussion and Conclusion

Patients with inflammatory bowel disease are at increased risk of development of skin cancer compared to the general population, independent of the use of immunosuppressants [2, 3]. Thus, this questionnaire aimed to assess IBD patients' actual and perceived risk factors for and knowledge of skin cancer as well as general skin protection strategies. Overall, we found that IBD patients are well informed about skin cancer and are cognizant of worrisome skin features. Despite this insight, they often practice suboptimal skin protection and prevention. According to the social-cognitive theory of health behavior, knowledge of risk alone is insufficient for behavior change; patients also require the perception of personal risk and skills training to change their habits [17]. This study highlights a potential opportunity for health professionals to teach and reinforce skin cancer prevention in IBD.

Other than treating the underlying inflammatory state of IBD, many of the factors that place these patients at additional risk of skin cancer development, including effective IBD treatments and early age at diagnosis, are not easily modifiable. However, similar to the general population, behavioral changes, such as avoidance of sun exposure and tanning beds and skin protection with use of sunscreen and protective clothing, can prevent or decrease the risk of skin cancer. One's perceived risk of skin cancer encourages these protective behaviors. On the contrary, a lack of awareness of skin cancer risk serves as a barrier to adoption of protective behaviors [14]. Overall, we found that IBD patients do have a modest understanding of skin cancer and are aware of worrisome signs and risk factors for skin cancer occurrence. In addition, our patient cohort does not report significant sun exposure. However, despite their knowledge and awareness, our patient population does not practice optimal sun protection or prevention strategies if they were to find a new mole.

Furthermore, when asked if IBD and its treatment increase one's skin cancer risk, 61% of patients agreed with this statement. Those with exposure to immunomodulators, which have been shown to increase the risk of skin cancer, did score higher for this variable. A proportion of our IBD population still does not recognize that their IBD increases their personal risk of skin cancer. Individuals adopt protective behavior (such as sun protection) if they perceive a health threat (such as skin cancer) [18, 19]. According to the health behavior model, if IBD patients do not perceive a personal risk of skin cancer development, they may not adopt effective skin protection behaviors [18]. Thus, it is our role as clinicians to discuss this topic with our patients. Furthermore, patients must understand that the proposed behavioral changes can mitigate this threat and that they can overcome barriers to achieving this [17, 18]. Accordingly, this highlights the importance of not only teaching patients about their own risk but also providing them with ways to minimize this risk with methods to protect oneself from the sun and its negative effects.

Moreover, we found that patients with a personal and family history of skin cancer tend to address worrying skin lesions with more vigilance and practice better sun protection than those without such histories. This further highlights that one's perceived risk of skin cancer development motivates one to adopt appropriate skin protection strategies. We found that females practice more appropriate sun protection and are better informed about skin cancer risk factors and worrisome skin features, which is consistent with prior studies [11]. Previous reports have found that young age is associated with high-risk behavior for skin cancer development, less knowledge about skin cancer, and increased incidence of skin cancer, especially melanoma [3, 11–14]. We found that patients over 40 years of age are more cognizant of when to seek medical attention for worrisome skin features. IBD patients often present in early adulthood, thus increasing the cumulative risk of skin cancer development. Specifically, younger men with Crohn's disease have an increased risk of nonmelanoma skin cancer; therefore, this is a particularly important group to target [3].

Our study has a few limitations. There is inherent reporter bias associated with the study design. The questionnaire format may both over- and/or underestimate skin cancer risk factors and skin practices. The participatory nature inherent in a research questionnaire leads to selection bias. Furthermore, given the anonymous nature of the survey, a diagnosis of IBD was self-reported and not confirmed via chart review. In addition, this study is limited by a sample primarily recruited in GI clinics at one large academic medical center. Thus, the study population may not be representative of all patients with inflammatory bowel disease. In addition, questionnaire items were compiled based on prior literature and skin cancer surveys; however, this specific survey has not been validated. Furthermore, some findings were statistically significant using a *P* value of <0.05 instead of 0.01; thus there is the possibility of a type I statistical error.

Our study shows that the majority of survey participants did recognize the connection between skin cancer and IBD. Comparatively, quality improvement studies have found even lower vaccination rates in the IBD population. Encouragingly, several studies have demonstrated remarkable improvements in vaccination rates with implementation of educational programs and increased access to vaccines [20–22]. As in vaccination studies, physician recommendations for skin cancer prevention may not be sufficient for behavioral change. Our study demonstrates that despite general knowledge about skin cancer and an overall understanding that they are at increased risk of skin cancer development, our IBD population continues to practice suboptimal skin protection. Educational modules may be necessary for IBD patients to further understand their personal risk of skin cancer development and to provide more information regarding appropriate preventive and protective behaviors. According to the social-cognitive theory of health behavior, in addition to knowledge of risk, patients also require guidance in tools to minimize this risk. Given the young age at IBD diagnosis, there is ample opportunity to intervene, educate, and reduce the cumulative risk of skin cancer development in this population.

This study supports the hypothesis that primary prevention of skin cancer in the IBD population is suboptimal and emphasizes a potential opportunity for healthcare professionals to highlight this population's risk and reinforce skin cancer prevention strategies. Implementation for improvement includes clinical reminders at each visit and educational modules on proper skin protection techniques.

## Figures and Tables

**Table:** (a) Skin cancer prevention

Questions addressed	Answers (%)				
	Very unlikely	Unlikely	Neutral	Likely	Very likely
How likely are you to visit a doctor if you noticed a new mole?	3.1	14.7	21.5	33.1	27.6
How likely are you to ignore a new mole?	1.9	16.0	22.2	29.0	30.9
How likely are you to check your own skin for moles?	7.5	14.4	23.8	36.3	18.1
If you found a new mole, how likely are you to see your primary care doctor right away?	32.7	37.7	19.1	5.6	4.9
If you found a new mole, how likely are you to see a dermatologist right away?	12.8	23.2	17.7	22.0	24.4
If you found a new mole, how likely are you to tell a family member or friend?	14.0	15.9	16.5	34.8	18.9
If you found a new mole, how likely are you to bring it up at your next doctor's appointment?	5.6	11.7	9.9	40.7	32.1

**Table:** (b) Skin cancer awareness

Questions addressed	Answers (%)				
How worried would you be if a mole	Not at all worried (1) through very worried (5)				
	1	2	3	4	5

Had irregular borders	4.9	6.1	17.1	29.9	42.1
Was asymmetric	6.1	6.7	18.4	30.7	38.0
Had 2 or more colors	2.5	3.7	13.0	28.0	52.8
Is greater than 6 mm in diameter	1.2	2.5	16.0	25.8	54.6
Grew in size	1.8	0.6	8.0	23.3	66.3
Is painful	1.8	1.2	5.5	22.7	68.7
Itches	1.2	3.0	17.7	23.2	54.9
Bleeds	1.2	0.6	6.7	20.9	70.6

Which of the following factors increase melanoma risk?	Percent (*N* = # of responses)				

None	0% (0)				
Having lots of moles	46% (76)				
Particular diets	10% (17)				
Family history of skin cancer	77% (126)				
Fair complexion	70% (114)				
Alcohol use	9% (14)				
Sunburns	80% (131)				
Prolonged sun exposure	79% (130)				
Smoking	30% (50)				
Blue eyes	26% (42)				
Green eyes	13% (22)				
Red hair	26% (42)				
Fair hair	29% (47)				
Immunosuppression	58% (95)				
All of the above	24% (40)				

**Table:** (c) Skin cancer knowledge

Questions addressed	Answers (%)	
	True	False
Skin cancer risk can be minimized by avoiding sun and using adequate sun protection	97.0	3.0
Skin cancer can be healed without treatment	1.8	98.2
Skin cancer can be cured if treated early	7.3	92.7
Skin cancer can lead to death if not treated	1.2	98.8

**Table:** (d) Skin protection

Questions addressed	Answers (%)				
	Never	Rarely	Occasionally	Frequently	Always
I use sunscreen if I am going to be outside for more than 20 minutes	4.9	18.3	31.1	28.7	17.1
I use SPF 30 or higher	4.3	9.3	18.0	29.8	38.5
I use sunscreen if it is cloudy outside	28.4	28.4	24.1	14.8	4.3
I reapply sunscreen after swimming or heavily perspiring	13.5	16.0	25.8	27.6	17.2
I use sunscreen while on vacation or holiday	3.0	6.7	17.7	37.2	35.4
I wear protective clothing in the sun	9.9	19.3	40.4	25.5	5.0
I use an umbrella to protect me from the sun	57.7	22.1	11.7	5.5	3.1
I wear a hat outside	20.5	19.3	36.0	18.0	6.2

**Table:** (e) Sun exposure

Questions addressed	Answers (%)				
	Never	Rarely	Occasionally	Frequently	Always

I spend more time outdoors than indoors	69.3	18.4	6.7	3.7	1.8
I try to get a suntan when outside	4.3	14.0	29.9	28.0	23.8
I spend a lot of time outdoors due to my occupation	1.3	10.0	42.5	35.0	11.3

	0		1-2	3-4	>5

How many sunny/beach/outdoor vacations have you been on in the last 5 years?	15.5		44.0	40.5	0.0

	Never			Current/prior use	

Have you ever used a tanning bed?	59.1			40.9	

	<10	10–25	25–50	50–100	>100

How many times have you used a tanning bed?	78.7	4.3	4.9	12.2	0.0

**Table 2:** Patient demographics.

Demographics	Percent of participants (*N*)
Gender	
Male	37% (61)
Female	63% (103)
Race	
White	94% (153)
Black	3% (5)
Multiple	2% (3)
Asian	1% (2)
Ethnicity	
Hispanic	2% (3)
Non-Hispanic	98% (161)
Married	57% (93)
College degree or higher	81% (132)
Employed full time	66% (108)
>$50,000 annual income	69% (111)
Private insurance	85% (139)
Smoking history	30% (48)
IBD diagnosis	
Crohn's disease	67% (109)
Ulcerative colitis	31% (51)
Indeterminate colitis	2% (4)
IBD surgery history	43% (71)
Current IBD treatment	95% (156)
Patient recruitment source	
Provider	75% (122)
Email	23% (37)
Other	3% (5)

**Table 3:** Patient skin cancer information.

	Percent of participants (*N*)
Skin biopsy history	43% (70)
Skin cancer history	12% (19)
Skin cancer type	
Basal cell	7% (12)
Squamous cell	2% (4)
Melanoma	1% (1)
Multiple types	4% (6)
First-degree relative skin cancer	34% (55)
Number of bad sunburns	
Never	6% (10)
1-2 times	31% (50)
3+ times	62% (102)
Unknown	1% (1)
Number of blistering sunburns	
Never	14% (23)
1-2 times	29% (48)
3+ times	14% (23)
Unknown	43% (70)
Skin exam frequency	
Less than once per year	30% (49)
Once per year	35% (57)
More than once per year	10% (17)
Never	24% (40)
Sees a dermatologist	70% (114)
Dermatologist referral source	
Gastroenterologist	13% (21)
Other specialists	2% (2)
Primary care	17% (27)
Self	51% (84)
Friend/family	2% (2)
Unknown	17% (28)

**Table 4:** Mean differences for skin cancer variables by various demographics and treatment.

	Scores				
	Protection (out of 32)	Exposure (out of 24)	Prevention (out of 28)	Awareness (out of 40)	Knowledge (out of 4)
Gender					
Male	14.53	6.49	15.52	32.98	2.95
Female	16.94^*∗*^	5.96	16.64	35.16^*∗*^	2.92
Immunomodulator treatment					
Currently treated	16.09	6.10	16.54	35.00	2.98
Previously treated	16.54	6.12	16.44	33.92	2.93
Never treated	15.58	6.23	15.82	34.14	2.90
Biologic treatment					
Currently treated	16.05	5.98	16.17	33.36	3.01^*∗∗*^
Previously treated	16.19	6.40	16.55	34.34	2.91
Never treated	16.08	6.19	16.16	35.45	2.85
Personal history of skin cancer					
No	15.33	6.05	15.75	34.03	2.92
Yes	21.47^*∗∗∗*^	6.91	20.56^*∗∗∗*^	36.81	3.00
Family history of skin cancer					
No	14.03	6.13	15.30	33.59	2.92
Yes	18.46^*∗∗∗*^	6.36	17.52	35.43	2.96

^*∗*^ *P* ≤ 0.03 , ^*∗∗*^ *P* ≤ 0.01, and  ^*∗∗∗*^ *P* ≤ 0.001 compared via *t*-tests.

**Table 5:** Patient responses to the following question: “Are you at increased risk of having skin cancer because of your IBD and the medications that you take for the treatment of IBD?”

Responses	Percent of participants (*N*)
Strongly agree	23.6% (39)
Agree	37.6% (62)
Neutral	33.9% (56)
Disagree	0.6% (1)
Strongly disagree	3.6% (6)
